# New WHO global air quality guidelines help prevent premature deaths in China

**DOI:** 10.1093/nsr/nwac055

**Published:** 2022-03-23

**Authors:** Tao Xue, Guannan Geng, Xia Meng, Qingyang Xiao, Yixuan Zheng, Jicheng Gong, Jun Liu, Wei Wan, Qiang Zhang, Haidong Kan, Shiqiu Zhang, Tong Zhu

**Affiliations:** Institute of Reproductive and Child Health / National Health Commission Key Laboratory of Reproductive Health and Department of Epidemiology and Biostatistics, School of Public Health, Peking University, China; State Key Joint Laboratory of Environmental Simulation and Pollution Control, School of Environment, Tsinghua University, China; School of Public Health, Key Laboratory of Public Health Safety of the Ministry of Education, and Key Laboratory of Health Technology Assessment of the Ministry of Health, Fudan University, China; State Key Joint Laboratory of Environmental Simulation and Pollution Control, School of Environment, Tsinghua University, China; Center of Air Quality Simulation and System Analysis, Chinese Academy of Environmental Planning, China; College of Environmental Sciences and Engineering, Peking University, China; School of Energy and Environmental Engineering, University of Science and Technology Beijing, China; Clean Air Asia, China; Ministry of Education Key Laboratory for Earth System Modeling, Department of Earth System Science, Tsinghua University, China; School of Public Health, Key Laboratory of Public Health Safety of the Ministry of Education, and Key Laboratory of Health Technology Assessment of the Ministry of Health, Fudan University, China; College of Environmental Sciences and Engineering, Peking University, China; College of Environmental Sciences and Engineering, Peking University, China

## Abstract

The World Health Organization has issued new air quality guidelines (AQG). Based on 2020 data, achieving the new AQG for PM2.5 could prevent an additional 285,000 chronic deaths and 13,000 acute deaths, across China, compared with the previous AQG. The new AQG can better protect health but cannot be achieved without coordinated air-pollution-control and climate-mitigation efforts.

The air quality guidelines (AQGs) issued by the World Health Organization (WHO) in 2005 are key references for the National Ambient Air Quality Standards (NAAQSs) in China. On 22 September 2021, the WHO issued new AQGs (NAQGs) [1], which propose more stringent targets to control exposure to multiple air pollutants, including fine particulate matter (PM_2.5_) and ozone (O_3_), to which, in 2019, 451 million premature deaths, globally, were attributable [2].

According to the AQGs, to protect public health, PM_2.5_ should be below 10 μg/m^3^ for annual average levels and below 25 μg/m^3^ for 24-h average levels. Informed by mounting evidence after the release of the 2005 AQGs, the NAQGs annual and 24-h average PM_2.5_ limits have been reduced to 5 and 15 μg/m^3^, respectively. In addition, the AQGs only consider short-term O_3_ exposure, with a recommended maximum daily 8-h average (MDA8) of 100 μg/m^3^. The NAQGs additionally introduced a peak-season average O_3_ limit of 60 μg/m^3^ to address the risk of chronic O_3_ exposure. These updates reflect the progress of research concerning the health effects of air pollutants, particularly the impacts of low-concentration PM_2.5_ exposure and long-term O_3_ exposure [3].

In China, during 2020, both PM_2.5_ and O_3_ annual averages exceeded the AQGs and NAQGs for the whole population. In particular, the 24-h PM_2.5_ average exceeded the AQGs and NAQGs for 75.4% and 51.1% person-days (out of 14 million persons × 365 days, i.e. the total time-at-exposure for the whole population), respectively. Moreover, the O_3_ MDA8 exceeded the AQGs/NAQGs for 36.9% person-days. Detailed population-weighted exposure distributions (2013–2020) are documented in Supplementary Fig. S1.

Based on the state-of-the-art datasets (Supplementary Text: Model inputs) and methods (Supplementary Text: Risk assessment models) [2,4–8], including those adopted by the Global Burden of Diseases 2019 assessment (GBD19), we quantified the number of avoidable deaths under different target-achievement scenarios (Table S1) compared with the measured pollution level of PM_2.5_ or O_3_ in a given year between 2013 and 2020. For instance, based on long-term PM_2.5_, the type of exposure that contributes to the most estimated deaths, and using 2020 levels, we found that achieving the NAQGs, AQGs and NAAQSs would prevent 1 215 000 (95% confidence interval [CI]: 1 129 000–1 302 000), 941 000 (874 000–1 008 000) and 116 000 (104 000–126 000) premature deaths, respectively (Fig. 1a). Thus, 96% (89.3%–100%) of long-term PM_2.5_ deaths would be avoided by achieving the NAQGs. The short-term PM_2.5_, long-term O_3_ and short-term O_3_ results are documented in Supplementary Fig. S2.

Meeting the WHO guidelines is the ultimate goal for actions concerning air quality control; to facilitate pathways toward this goal, the WHO proposed interim targets (ITs). In the new guidelines, AQGs for PM_2.5_ have been added as an additional IT (i.e. IT-4). By comparing our assessment results, we further quantified the additional avoidable deaths (AADs) of achieving a more stringent target compared with a less stringent one (Fig. 1a). Each step we take towards achieving a more stringent WHO IT or NAQGs target will help prevent more deaths attributable to long-term PM_2.5_. Of the steps required to reduce air pollution, we were particularly interested in the health benefits of the ‘last-step’ scenario, that is, going from achieving the AQGs to achieving the NAQGs. Based on 2013 data for long-term PM_2.5_, the last-step scenario yielded 223 000 (202 000–239 000) AADs; this increased to 285 000 (251 000–300 000) AADs when using 2020 data (Fig. 1b, top). These findings are the result of the non-linear exposure–response function of PM_2.5_; at lower PM_2.5_ concentrations, the per-unit PM_2.5_ increase is associated with a higher relative risk, suggesting that more health benefits can be achieved from a per-unit PM_2.5_ reduction (Fig. S3). Such benefits encourage us to aim for more stringent PM_2.5_ targets to improve air quality in China. The spatial distributions of the last-step AADs were impacted by population density and vulnerability; there were more AADs in more populous, less polluted regions (Fig. 1c). Thus, improving PM_2.5_ levels to meet the standards of the NAQGs as opposed to the AQGs will confer more health benefits in megacities, such as Shanghai. Short-term PM_2.5_ AADs showed similar trends and are documented in Supplementary Figs S4 and S5.

**Figure 1.**
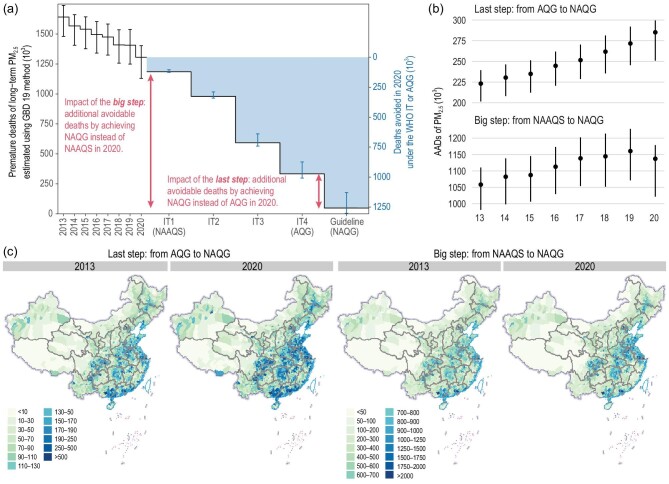
Quantification and prevention of premature deaths resulting from long-term PM_2.5_ exposure as a result of achieving different clean air targets. (a) Premature deaths resulting from long-term PM_2.5_ in China at current levels (2013–2020) and projected reductions under the World Health Organization (WHO) interim target (IT) or air quality guidelines (AQGs) scenarios. The double-arrowed lines show the additional avoidable deaths (AADs) resulting from the last-step and big-step scenarios based on 2020 PM_2.5_ levels; note that the AADs varied depending on different PM_2.5_ baselines. (b) AADs attributable to long-term PM_2.5_ exposure by achieving the new AQGs (NAQGs) compared to the previous AQGs (last-step scenario) or National Ambient Air Quality Standards (NAAQSs) of China (big-step scenario) under different PM_2.5_ baselines, from 2013 to 2020. (c) County-level maps of the spatial distribution of AADs under the last-step and big-step scenarios for China for 2013 and 2020. In panels (a) and (b), the error bars represent 95% confidence intervals, produced from Monte Carlo simulations. In panel (c), data for the Taiwan Province and some small islands are not available. Review drawing number: GS(2022)1347.

China included PM_2.5_ in the NAAQSs for the first time in 2012 based on IT-1. The NAAQSs for PM_2.5_ played a critical role in defining the targets of subsequent clean air actions in China (2013–2017 and 2018–2020). These actions rapidly reduced PM_2.5_ pollution [8–10] to levels close to the NAAQSs for both long- and short-term PM_2.5_, and prevented a significant number of premature deaths [8,9] (Fig. 1b). To further assess the need for enhanced air pollution control targets in China, we calculated the AADs of another scenario termed the ‘big step’, that is, going from achieving the NAAQSs to achieving the NAQGs. From 2013 to 2020, the number of AADs first increased and then slightly decreased from the peak in 2019 (Fig. 1b, bottom). One explanation is that in China, an increasing number of regions have achieved NAAQSs PM_2.5_ levels (Figs S1 and S4). In 2020, the big-step scenario additionally avoided 1 137 000 (1 021 000–1 179 000) deaths, accounting for 90.4% (89.8%–91.2%) of all premature deaths avoided by achieving the NAQGs.

Uncertainties embedded in the NAQGs and the relevant risk assessments, including ours, should be noted and addressed in future studies. First, the NAQGs cannot be applied to air pollutant mixtures, and premature deaths associated with different single-pollutant exposures are not additive. Recently, a few methods (e.g. Bayesian kernel machine regression) have been developed to estimate the health effects of multi-pollutant mixtures [11], and should be applied in future epidemiological studies. Second, the NAQGs were based on epidemiological findings from sampled individuals, and assumed to be applicable to the whole population. Whether the NAQGs are applicable to the Chinese population should be further examined based on Chinese studies of health effects of low-concentration exposure. Third, the toxic components of air pollutant mixtures have not been clearly distinguished. In the NAQGs report, the WHO stated the need to assess specific toxicants, including ultrafine particles, black carbon and dust particles, because of a scarcity of exposure data from large population studies. Therefore, exposure assessment methods based on a fusion of multiple data inputs (e.g. monitoring data of low-cost sensors, chemical transport modeling outputs and satellite remote sensing measurements) should be relied on in the future. Finally, the AQGs and ITs were developed from risk assessments, which could be limited due to the spatial resolution of exposure data (e.g. causing potential misclassification of exposure in megacities using our 10 × 10 km data), ignored fluctuations in population (e.g. migration), and uncertainties embedded in baseline mortalities and exposure–response functions. In risk assessments used to develop environmental standards, such uncertainties should be comprehensively quantified, as in the present study (Supplementary Text: Analysis of uncertainty). Moreover, uncertainties should be considered when planning relevant policies in China. A quantitative cost–benefit analysis may help identify the trade-off between acting sooner and waiting to act after the uncertainties are resolved.

Many improvements are needed to achieve the NAQGs in China, which were promoted under the recently proposed targets to mitigate climate change. The strategy to achieve carbon neutrality in 2060 has been reported to reduce the PM_2.5_ concentration to 8 μg/m^3^ [12], which meets the AQGs but falls short of the NAQGs. Driven by both the carbon neutrality and NAQGs goals, we anticipate a rapid reduction in air pollution and greenhouse gas emissions. Additionally, based on an integrated assessment from 2015 to 2050, a recent study [13] showed that without ambitious climate goals, stringent clean-air policies alone cannot substantially reduce premature deaths attributable to PM_2.5_ in China. Therefore, a coordinated effort to align air pollution control policies and climate mitigation policies is warranted to maximize public health and sustainable development benefits and to minimize socio-economic costs. Protecting public health, the core driver of the NAQGs, puts the focus on the current population and is closely related to sustainable development goals and economic growth; meanwhile, achieving carbon neutrality, which increases resilience and sustainability, will determine current and future well-being. Such coordinated efforts require not only emission control measures that offer multiple benefits (e.g. electrification [14]) but also an optimized timeline of actions, considering future trajectories of other influencing health factors (e.g. aging [9,13]) and feedbacks among environmental, health and socio-economic systems.

## Supplementary Material

nwac055_Supplemental_File
