# Supplementary material for: New WHO global air quality guidelines help prevent premature deaths in China
Source: Natl Sci Rev. 2022 Mar 23;9(4):nwac055. doi: 10.1093/nsr/nwac055 (PMC9084177; doi:10.1093/nsr/nwac055)
Supplement: nwac055_Supplemental_File [file nwac055_supplemental_file.docx]

**New WHO Global Air Quality Guidelines Help Prevent Premature Deaths in China**

Tao Xue^1^, Guannan Geng^2^, Xia Meng^3^, Qingyang Xiao^2^, Yixuan Zheng^4^, Jicheng Gong^5^, Jun Liu^6^, Wei Wan^7^, Qiang Zhang^8^, Haidong Kan^3^, Shiqiu Zhang^5^, Tong Zhu^5,^*

1. Institute of Reproductive and Child Health / Ministry of Health Key Laboratory of Reproductive Health and Department of Epidemiology and Biostatistics, School of Public Health, Peking University, Beijing, China;
2. State Key Joint Laboratory of Environmental Simulation and Pollution Control, School of Environment, Tsinghua University, Beijing, China;
3. School of Public Health, Key Lab of Public Health Safety of the Ministry of Education, & Key Lab of Health Technology Assessment of the Ministry of Health, Fudan University, Shanghai, China;
4. Center of Air Quality Simulation and System Analysis, Chinese Academy of Environmental Planning, 100012 Beijing, China;
5. College of Environmental Sciences and Engineering, Peking University, Beijing, China;
6. School of Energy and Environmental Engineering, University of Science and Technology Beijing, Beijing 100083, China;
7. Clean Air Asia, Beijing, China;
8. Ministry of Education Key Laboratory for Earth System Modeling, Department of Earth System Science, Tsinghua University, Beijing China.

* Corresponding to Tong Zhu (tzhu@pku.edu.cn)

**Supplemental Text: Methods**

We first conducted a series of risk assessments concerning the health impacts of PM_2.5_ and O_3_ using the different air quality standards, the classical method, and the best available data. Then, we quantified the AAD using the NAQG, compared with the AQG or NAAQS.

***Model inputs***

The daily gridded PM_2.5_ and O_3_ concentrations were obtained from TAP (http://tapdata.org.cn/). The details of the exposure assessment models have been published previously ^1^ and are summarized here. The PM_2.5_ predictions incorporated in situ PM_2.5_ observations, community multiscale air quality simulations, and satellite measurements, using a two-stage model. The first-stage model captured the high PM_2.5_ episodes, using a synthetic minority oversampling technique. The second-stage model calibrated the community multiscale air quality simulations using PM_2.5_ measurements with first-stage results, satellite measurements, and auxiliary variables. This model produced daily PM_2.5_ concentrations with complete spatiotemporal coverage in a regular 0.1 × 0.1° grid across China. Model performance was evaluated by out-of-bag cross-validation, and the model prediction results and in situ observations showed good agreement (R^2^ = 0.77–0.91). A previous study ^1^ also showed that the TAP product could capture the temporal trends in PM_2.5_. Thus, compared with other sources, the TAP model is advantageous for its high-quality inputs (including emission inventories) and incorporation of a well-established gap-filling method ^2^.

The MDA8 O_3_ concentrations were estimated using a three-stage random forest model of inputs similar to PM_2.5_ ^3^. This model combined in situ observations with community multiscale air quality simulations, ozone monitoring instrument satellite O_3_ profiles (PROFOZ; v0.9.3, level 2), and auxiliary variables (e.g., meteorological parameters, the normalized difference vegetation index, and nightlight data). Details regarding the algorithm and its performance were documented in our previous publication. The long-term trends estimated using the model showed good consistency with those estimated from monitoring data (R^2^ = 0.57 at a county-level scale). In this study, the model was updated as follows. First, the previous product included the period from 2013 to 2017; the analysis period was extended to 2020 in this study. Second, we calibrated all predictions to the new reference state (298.15 K, 101.325 kPa), with reference to the protocol launched on September 1, 2018 (HJ 654–2013). Third, we replaced the meteorological variables simulated by community multiscale air quality with meteorological variables obtained from the MERRA-2 reanalyzed product. The latter product improved overall model performance. These predictions were in good agreement with monitored O_3_ concentrations (R^2^ = 0.84) ^4^. The spatiotemporal resolution of the O_3_ product was the same as the spatiotemporal resolution of the PM_2.5_ product.

**Population distribution and baseline mortality**

We obtained gridded population data from the LandScan Global Population Database from 2003 to 2019 ^5^. We assumed that the population changed linearly, then estimated the population distribution in 2020 on the basis of the 2017–2019 LandScan data. We also scaled the annual LandScan population to be consistent with the national sum population reported by the China Statistical Bureau. The national age- and gender-specific baseline mortalities for different endpoints and calendar years were obtained from the GBD database (<https://vizhub.healthdata.org/gbd-compare/>). The age- and gender-specific structures of the Chinese population by year were also obtained from the GBD database. The long-term exposure-response functions for PM_2.5_ and O_3_ were obtained from the results of the GBD19; the short-term functions were obtained from nationally representative time-series studies that incorporated 272 cities in China.

**Risk assessment models**

In GBD studies, ambient PM_2.5_ or O_3_ exposure is assumed to increase the probability of premature death among adults by increasing the incidence or mobility of chronic diseases, including stroke, ischemic heart disease, chronic obstructive pulmonary disease, lung cancer, and diabetes. To conduct exposure–response functions, integrated from many individual epidemiological studies on the health effects of air pollution, the GBD model assumes that the relative risks of mortality and incidence are equal ^6^. Therefore, results from the model also reflect the risk of fatality resulting from increased morbidities associated with air pollution exposure. Notably, the GBD model only considers the risk of fatality (measured as the number of premature deaths or the loss of life expectancy) but ignores disability caused by attributable diseases (e.g., stroke), even though recent studies have shown that PM_2.5_ exposure can contribute to physical function disability ^7^. Thus, although the method developed by the GBD19 assessment ^8^ offers the most current approach to evaluate the health impacts of ambient air pollution, it has some limitations.

We applied the GBD model in this study. The risk assessment model for each exposure-outcome pair was uniformly expressed by the following equation:

AF_s,t,k_ = max[1 – f_k_(C_0_) / f_k_(C_s,t_), 1]; D_s,t,k_ = AF_s,t,k_ * P_s,t,k_ * B_s,t,k_ …… (1)

where s, t, and k denote the indices for the geographic unit, temporal unit (day or year for short- or long-term assessment, respectively), and subpopulation index (by sex and age); P_s,t,k_, B_s,t,k_, AF_s,t,k_, and D_s,t,k_, denote the population size, baseline mortality rate, fraction, and number of premature deaths attributable to the exposure-outcome pair within a group of people defined by s, t, and k, respectively; C_s,t_ denotes the exposure concentration; C_0_ denotes the counterfactual concentration, which was set according to the NAAQS, AQG, NAQG, or TMREL (used in the GBD19); and f_k_() denotes the exposure-response function, predicting the relative risk of premature deaths considering a specific exposure level. Here, D_s,t,k_ was interpreted as (1) premature deaths, if the C_0_ was assumed to be the TMREL or the threshold concentration (i.e., the effect of any concentration less than C_0_ is zero), or (2) avoidable premature deaths by achieving the C_0_ target (i.e., the counterfactual scenario, where all concentrations [C_s,t_ > C_0_] above are reduced to C_0_, and the rest [C_s,t_ ≤ C_0_] remain unchanged). Because determination of the true threshold concentration is complex and exceeds the scope of this study, we utilized the second interpretation of D_s,t,k_. In accordance with the WHO guidelines, we considered four exposure terms: long-term PM_2.5_, long-term O_3_, short-term PM_2.5_, and short-term O_3_. We applied cause-specific mortalities for the first term and total mortality for the remaining three terms as the corresponding outcomes. All assessments were evaluated at the county level. We used equation (2) to calculate the national level sums, considering a specific C_0_:

[D_t_ | C_0_] = ∑ _s,k_ D _s,t,k_, C_0_ = GBD19, NAQG, AQG, or NAAQS …… (2).

**Uncertainty analysis**

Uncertainties embedded in the above assessments were quantified using the Monte Carlo approach. We considered potential input-related errors, including baseline mortality rates, population structures, exposure-response functions, exposure concentrations, population sizes, and GBD19. The first three inputs were obtained from direct estimates with reported 95% CIs; their uncertainties were simulated by the corresponding normal distributions. GBD19 was assumed to exhibit a uniform distribution. Standard errors of the exposure estimates were estimated by cross-validation assessments and inputted into the normal distributions to evaluate the uncertainties embedded in the PM_2.5_ or O_3_ concentrations. Since the cross-validation compared the gridded estimates with the in situ (point-level) observations, it not only characterized the uncertainties resulting from the modeling procedures but also captured those attributable to spatial misalignments or exposure misclassifications; for instance, within a 10 × 10 pixel area, the air pollution concentration can vary substantially and should not be quantified using a single predicted value, particularly in megacities. Population size estimated by the LandScan product was evaluated using city-level statistical yearbooks. We utilized a log-linear regression of the yearbook records against the city-level sums of the scaled LandScan estimates, then used the asymptotic normal distribution to simulate the uncertainty embedded in the LandScan products.

***Derivation and interpretation of additional avoided deaths***

Based on the risk assessment results, we derived the number of AAD using the following equation:

[AAD_t_ | C_0_ ~ C_1_] = [D_t_ | C_0_] – [D_t_ | C_1_], C_0_ < C_1_ …… (3),

where C_0_ and C_1_ denote two different counterfactual concentrations, while [AAD_t_ | C_0_–C_1_] quantifies the difference between the estimates of avoidable premature deaths given C_0_ and given C_1_. AAD can be directly interpreted as the number of additional premature deaths that can be prevented by achieving C_0_ (e.g., the NAQG), instead of the less stringent target C_1_, (e.g., the AQG). To evaluate the WHO revision, C_0_ was set as the NAQG and C_1_ was set as the AQG or NAQG. AAD can be used as a metric to reflect the degree of encouragement to improve the air quality target from C_1_ to C_0_. When other inputs are fixed, AAD is determined by the change in AF related to different counterfactual concentrations (Figure S2) and varies with the current exposure level (C). AAD is zero for C < C_0_, suggesting that the target improvement results in no difference because the actual air quality has already achieved the target. For C >> C_1_, AAD is similar to C, suggesting less incentive for target improvement because C_1_ should be achieved first. For C close to C_1_, AAD peaks at C_1_, suggesting an incentive for target improvement. Under this scenario, C_1_ will be achieved soon or has recently been achieved; thus, it should be updated to a stringent target, C_0_. In particular, AAD is maximized when C is equal to C_1_, which indicates the optimal time for target replacement with a more stringent value. AAD depends on current exposure level and is positively associated with population vulnerability (e.g., advanced age structure or worse baseline mortality) or population density. For a population with increased vulnerability or lived-in populous areas, an enhanced air quality target is encouraged; this also illustrates the policy implications for AAD. In this study, we calculated [AAD | NAQG–AQG] and [AAD | NAQG–NAAQS] for different counties and calendar years, thus exploring whether the corresponding improvement in air quality target was warranted. The AAD analyses were only performed on PM_2.5_ because it is the major contributor to the disease burden.

**Supplemental Table**

Table S1 Different theoretical minimum risk exposure levels used in the risk assessment model.

|  | O_3_ | | PM_2.5_ | |
| --- | --- | --- | --- | --- |
|  | Long-term | Short-term | Long-term | Short-term |
| GBD19 | 29.1–35.7 ppb | | 2.4–5.9 µg/^3^ | |
| NAAQS | NO | 160 µg/m^3^ | 35 µg/m^3^ | 75 µg/m^3^ |
| WHO AQG | NO | 100 µg/m^3^ | 10 µg/m^3^ | 25 µg/m^3^ |
| NAQG | 60 µg/m^3^ |  | 5 µg/m^3^ | 15 µg/m^3^ |

**Supplemental Figures**

Figure S1 Population-weighted cumulative probability distributions of PM_2.5_ and O_3_ from 2013 to 2020 in China. The cumulative probability curves were derived from a combination of gridded concentrations and population distributions, as described in the Supplemental Text: Methods. For a given year, the gridded populations were used as weights to calculate the probabilities of the gridded concentrations. The results showed that most of the population was exposed to PM_2.5_ or O_3_ above the AQG or NAQG in the long-term scenario; compared with China’s current standards, 17.9% of the total population was below the NAAQS in 2013, and the fraction increased to 60.6% in 2020. For short-term PM_2.5_ exposure in 2013, 6.0%, 19.3%, and 71.3% of all exposures in person-years were below the NAQG, AQG, and NAAQS, respectively; the fractions increased to 24.6%, 48.9%, and 93.1%, respectively, in 2020. For short-term O_3_ exposure in 2013, 74.8% and 99.2% of all exposures in person-years were below the AQG or NAAQS, respectively; the fractions decreased to 63.1% and 94.9%, respectively, in 2020 because of increased pollution. Our data show that achieving the enhanced WHO guideline (i.e., the NAQG) poses a new challenge for controlling long-term PM_2.5_ and O_3_ exposure in China.

**(a) (b)**

**(c) (d)**

Figure S2 Avoidable premature deaths in China as a result of achieving target PM_2.5_ and O_3_ levels based on data from 2013 to 2020. GBD19: theoretical minimum risk exposure level used in the global burden of disease assessment 2019; AQG: air quality guidelines issued by the World Health Organization in 2005 (WHO); NAQG: new AQG issued by the WHO in 2021; NAAQS: National Ambient Air Quality Standards of China. Although the absolute values were different, the estimates assuming all TMRELs had similar temporal trends. The number of long- and short-term deaths attributable to PM_2.5_ decreased rapidly from 2013 to 2020. The number of long- and short-term deaths attributable to O_3_ first increased from 2013 to 2019, then slightly decreased in 2020. These trends are consistent with previous findings. The underlying reasons have been discussed in previous analyses ^9, 10^, and thus exceed the scope of this study. In 2020, achieving the NAQG, AQG, or NAAQS for long-term PM_2.5_ would avoid 1,215,000 (1,129,000–1,302,000), 941,000 (874,000–1,008,000), or 116,000 (104,000–126,000) premature deaths, respectively; achievement of the three targets for short-term PM_2.5_ would avoid 47,000 (34,000–62,000), 34,000 (25,000–44,000), or 6000 (4000–8000) premature deaths, respectively. There is no AQG or NAAQS for long-term O_3_ exposure; achieving the NAQG would avoid 163,000 (75,000–237,000) premature deaths. The WHO did not update the guidelines for short-term O_3_ exposure. Achievement of the AQG or NAAQS would avoid 33,000 (19,000–48,000) or 4000 (2000–6000) deaths, respectively. These results suggest that the NAQG would structurally shift the estimated deaths attributable to O_3_. Long-term effects, rather than short-term effects, were the major contributors to premature death from O_3_. Figure 3 shows the reproduced results based on the methods used in the GBD19 study ^8^. Those results were comparable with the avoidable deaths after achieving the NAQG.

1. (b)

Figure S3 Illustration of long-term (a) and short-term (b) exposure–response functions for PM_2.5_, considering different theoretical minimum risk exposure levels. (a) Functions for lung cancer as an example; the curves were similar for other outcomes and thus are not shown here. (b) Functions for total mortality. The figure also shows reference probability distributions of PM_2.5_ exposure.

(a)

(b)

Figure S4 Additional avoidable deaths (AADs) attributable to long-term PM_2.5_ exposure by achieving the new WHO air quality guidelines (NAQG, revised in 2021) compared with the previous air quality guidelines (AQG, in 2005) or the National Ambient Air Quality Standards of China (NAAQS). (a) Absolute values of total AADs across China in 2013–2020. (b) AAD percentages of all premature deaths avoided by achieving the NAQG.

(a)

(b)

(c)

(d)

Figure S5 Spatial distributions of additional avoidable deaths (AADs) attributable to long- or short-term PM_2.5_ exposure as a result of achieving the new WHO air quality guidelines (NAQG, revised in 2021), instead of the previous air quality guidelines (AQG, in 2005) or the National Ambient Air Quality Standards of China (NAAQS). The ADDs were the result of a change in target achievement (a) from AQG to NAQG for long-term PM_2.5_, (b) from AQG to NAAQS for long-term PM_2.5_, (c) from AQG to NAQG for short-term PM_2.5_, and (d) from AQG to NAAQS for short-term PM_2.5_.

**References**

1. Geng G*, et al.* Tracking Air Pollution in China: Near Real-Time PM2.5 Retrievals from Multisource Data Fusion. *Environmental Science & Technology*, (2021).

2. Xiao Q*, et al.* Evaluation of gap-filling approaches in satellite-based daily PM2.5 prediction models. *Atmospheric Environment* **244**, 117921 (2021).

3. Xue T*, et al.* Estimating Spatiotemporal Variation in Ambient Ozone Exposure during 2013–2017 Using a Data-Fusion Model. *Environmental Science & Technology* **54**, 14877-14888 (2020).

4. Xiao Q*, et al.* Tracking PM2.5 and O3 Pollution and the Related Health Burden in China 2013–2020. *Environmental Science & Technology*, (2021).

5. Dobson JE, Bright EA, Coleman PR, Durfee RC, Worley BA. LandScan: a global population database for estimating populations at risk. *Photogrammetric Engineering and Remote Sensing* **66**, 849-857 (2000).

6. Burnett RT*, et al.* An integrated risk function for estimating the global burden of disease attributable to ambient fine particulate matter exposure. *Environmental Health Perspectives* **122**, 397-403 (2014).

7. Wang H*, et al.* Association between Ambient Fine Particulate Matter and Physical Functioning in Middle-aged and Older Chinese Adults: A Nationwide Longitudinal Study. *The Journals of Gerontology: Series A*, (2021).

8. Murray CJ*, et al.* Global burden of 87 risk factors in 204 countries and territories, 1990–2019: a systematic analysis for the Global Burden of Disease Study 2019. *The Lancet* **396**, 1223-1249 (2020).

9. Zhang Q*, et al.* Drivers of improved PM2.5 air quality in China from 2013 to 2017. *Proceedings of the National Academy of Sciences* **116**, 24463-24469 (2019).

10. Geng G*, et al.* Drivers of PM2.5 air pollution deaths in China 2002–2017. *Nature Geoscience* **14**, 645-650 (2021).
